# RADAR: Transforming Inpatient Psychiatry Ward MDT Meetings Through a Structured Communication Tool

**DOI:** 10.1192/bjo.2026.11443

**Published:** 2026-06-30

**Authors:** Umar Nasser, Jonathan Taylor, Ekene Okonji, Mark Pecover, Tope Forsyth

**Affiliations:** Surrey and Borders Partnership Trust, Surrey, United Kingdom

## Abstract

**Aims:**

Daily multidisciplinary team (MDT) meetings are essential for safe and effective inpatient psychiatric care. Across inpatient psychiatric wards within Surrey and Borders Partnership Trust (SABP), baseline audits using a Terms of Reference (TOR) self-assessment tool revealed low reliability in MDT processes, with only 53% adherence to core clinical discussion protocols. This highlighted significant variability in how risk, care planning and discharge planning were addressed. The aim of this QIP was for all wards to adhere to a 90% average using TOR self-assessment by the end of the study period.

**Methods:**

Applying the Model for Improvement as a structural framework, a diagnostic phase utilized Pareto analysis to isolate the most critical MDT omissions. Process mapping visualized current practices, while Failure Mode and Effects Analysis (FMEA) identified domains carrying the highest clinical risk. To generate change ideas, weekly workshops using TRIZ methodology were held with three pilot wards. From this, the structured conversation tool ‘RADAR’ was developed.

RADAR directs MDT conversations around five domains: Risk; Action Plans; Discharge Plans; Assign Tasks; Review Previous Plans. Open-ended verbal prompts using these domains ensure key clinical areas are addressed, i.e.: “What is the risk?”, “Are thecurrent action plans effective?”, “Where are we with discharge planning?”, “Let’s assign tasks”, “Let’s review yesterday’s plan”.

Following initial positive testing on one ward, RADAR was rolled out across seven wards, including PICU, General Adult and Old Age Psychiatry. Compliance data was gathered weekly for three weeks using a TOR self-assessment tool, with data monitored via Statistical Process Control (SPC) charts.

**Results:**

Seven wards implemented RADAR. Average compliance for individual wards ranged from 63% to 94.3%. The overall mean compliance across the study period was 79.3%, and by the third week, mean compliance reached 84.7%. While this fell short of the 90% target, it represented a sustained upward trend and a 31-percentage-point increase from the original 53% baseline. Following this QIP, RADAR was incorporated into the Business-As-Usual (BAU) protocols across SABP.

**Conclusion:**

RADAR was shown to be an effective tool for structuring clinical conversation, significantly improving compliance in areas such as risk and discharge planning. It facilitates a ‘closed loop’ by assigning tasks, and then reviewing tasks that were previously assigned each day. A memorable acronym that prompted open-ended questions likely aided its real-world effectiveness. Further research is required to assess its impact on Red-to-Green (R2G) bed days and its efficacy across wider clinical sub-specialties.

